# H_2_S Donors Reverse Age-Related Gastric Malfunction Impaired Due to Fructose-Induced Injury *via* CBS, CSE, and TST Expression

**DOI:** 10.3389/fphar.2020.01134

**Published:** 2020-07-24

**Authors:** Yaroslav Pavlovskiy, Antonina Yashchenko, Oksana Zayachkivska

**Affiliations:** ^1^ Physiology Department, Danylo Halytsky Lviv National Medical University, Lviv, Ukraine; ^2^ Histology, Cytology and Embryology Department, Danylo Halytsky Lviv National Medical University, Lviv, Ukraine

**Keywords:** hydrogen sulfide, donor, gastric mucosa, aging, fructose, oxidative stress

## Abstract

**Objective:**

Excess of fructose consumption is related to life-treating conditions that affected more than a third of the global population. Therefore, to identify a newer therapeutic strategy for the impact prevention of high fructose injury in age-related malfunctions of the gastric mucosa (GM) in the animal model is important.

**Methods:**

Adult and aged male rats were divided into control groups (standard diet, SD) and high fructose diet (HFD) groups; acute water immersion restraint stress (WIRS) was induced for evaluation of GM adaptive response and effects of testing the therapeutic potential of H_2_S-releasing compounds (H_2_S donors). Histological examination of gastric damage was done on hematoxylin-eosin stained slides. Cystathionine beta-synthase (CBS), Cystathionine gamma-lyase (CSE), and Thiosulfate-dithiol sulfurtransferase (TST) activities and oxidative index were assessed during exogenous administration of H_2_S donors: sodium hydrosulfide (NaHS) and the novel hybrid H_2_S-releasing aspirin (ATB-340). The results showed that HFD increased gastric damage in adult and aged rats. HFD-associated malfunction characterized by low activities of H_2_S key enzymes, inducing increased oxidation. Pretreatment with NaHS, ATB-340 of aged rats in the models of HFD, and WIRS attenuated gastric damage in contrast to vehicle-treated group (p < 0.05). The effect of ATB-340 was characterized by reverse oxidative index and increased CBS, CSE, and TST activities. In conclusion, H_2_S donors prevent GM age-related malfunctions by enhancement of CBS, CSE, and TST expression against fructose excess injury though reduction of oxidative damage.

## Introduction

The World Health Organization defined relation between excess fructose consumption and accelerated aging, which could be a factor for multi-morbidity and has numerous harmful effects (Lyons et al., 2016; Bektas et al., 2018; Miller et al., 2018). Oxidative stress is one of their molecular mechanisms for cellular survival, as well as damage, and accelerated aging that could be the result of the decline of integrated defensive molecular mechanisms (Franceschi et al., 2018a; Franceschi et al., 2018b; Cherkas et al., 2020). Therefore, to search for a novel physiologically based therapeutic strategy that enhances the natural mechanisms of cytoprotection and reverses the influence of aging and high fructose injury is the actual topic in modern pharmacology JL (Wallace et al., 2015; Engevik, 2018). It is known that age-related changes in the gastrointestinal mucosal defense formed increased gastric mucosa (GM) susceptibility to injuries and enhanced low-grade inflammation related to excessive oxidative stress (distress) toxic injury that have systemic character (Schultz et al., 2018; Tarnawski and Ahluwalia, 2018).

Hydrogen sulfide (H_2_S) system, the key endogenous signaling mediator that is operated by several enzymatic and non-enzymatic pathways, including catalytic activities of Cystathionine-β-synthase (CBS, EC 4.2.1.22), Cystathionine-γ-lyase (CSE, EC 4.4.1.1) and Thiosulfate-dithiol sulfurtransferase (belong to sulfurtransferase superfamily and rhodanese family, another name—thiosulfate reductase (glutathione-depend), TST, EC 2.8.1.5) is known for more than 15-years study. These key enzymes in H_2_S signaling operated in the cytosol (CBS, CSE) and mitochondria (CBS and TST) showed remarkable cytoprotective advances of their multifunctional abilities, as gas neurotransmitters, the mediator of vascular response, platelet adhesion, regulation of glucose metabolism, redox balance, and detoxification of intramitochondrial oxygen free radicals (Wallace et al., 2012; Fu et al., 2012; Szabo et al., 2014; Kanagy et al., 2017; Zhang et al., 2017). Latest studies have concluded that H_2_S is the remarkable activator of endothelial vasoactive agents, chemotaxis of immune cells and scavenger of active forms of oxygen species, neutralization of cytokines and autophagy, thus, there is a reason for creation of a pharmacological way to prevent accelerated aging and treat age-related disorders (Sharkey and Mawe, 2014; Pichette and Gagnon, 2016; Wang et al., 2017; Das et al., 2018). Since H_2_S is involved in the regulation of intracellular redox status, as well as signaling cascades of phosphorylation, apoptosis, involving mitochondrial K^+^ ATP-channels, it is possible to interpret such actions as a powerful antiradical resource (Nakajima, 2015; Mard et al., 2018). TST activity is important for mitochondrial oxidation and could be an effective marker of mitochondrial dysfunction in response to oxidative stress, but its role in the coupling of CBS, CSE expression in the H_2_S pathway of age-related changes in gastric cytoprotection against fructose injury is still unknown.

Since the defense reactions of people in advanced age are related with cross-talk of oxidative stress and vascular health, regulated by hypoxia-inducible factor (HIF) that triggered synthesis and release of vascular endothelial growth factor/vascular permeability factor (VEGF/VPF), and as the result of age-related tissue malfunction, which is the first stage of injury, and if not prevented or treated early, the next cellular stage will often induce tissue damage (Donnarumma et al., 2011; Arumugam and Kennedy, 2018). Thus, there is a reason for study of H_2_S donors—H_2_S-releasing compounds, as agents that could decrease the impact of aging on the susceptibility of GM to high fructose injury in laboratory models by exploring changes in expression of CBS, CSE, and TST.

**Figure 1:**
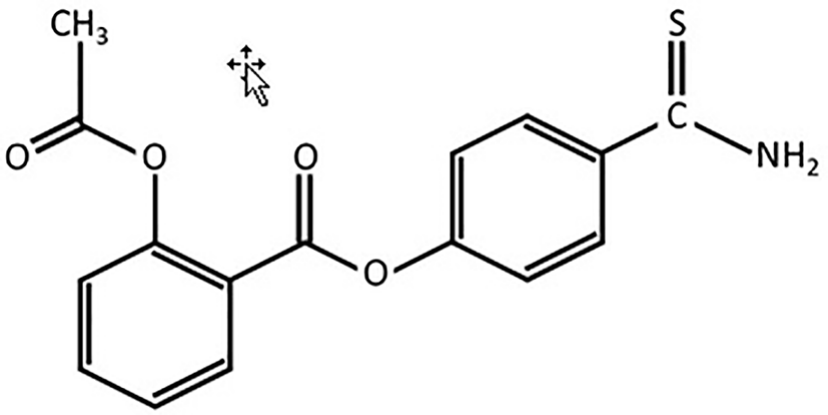
Chemical structure of 4-(5-thioxo-5H-1,2-dithiol-3-yl)phenyl 2-acetoxybenzoate(l)—H_2_S-releasing aspirin (ATB-340).

Present-day animal models of human diseases play an important role in the translational research that helps in identification and testing of compounds for age-related differences in cytoprotection (Verma et al., 2017; Wallace et al., 2018). Studies have shown that exogenous sodium hydrosulfide (NaHS) is the non-organic donor of synthesis of H_2_S and enhancer for catalytic activities of CBS and CSE (Wallace et al., 2007; Di Villa Bianca et al., 2011). Recently, it has been found that the synthesis of H_2_S from cysteine and cysteine with homocysteine affects glucose regulation and provides protection of vascular endothelium against oxidative stress and correction of hyperhomocysteinemia, which may be a predictor of accelerated aging and H_2_S signaling network is the source for reversion of vascular aging (Das et al., 2018; De Cabo and Diaz-Ruiz, 2020). Interestingly, H_2_S mediates the activity of arachidonic cycle and production of prostaglandins and recent advances in the creation of the latest hybrid H_2_S-releasing drugs, analogs of nonsteroidal anti-inflammatory drugs (NSAIDs, H_2_S-NSAIDs) or organic Lawesson’s reagent derivative, have shown ability to reverse decrementing side effects that arise from the digestive system, cardiovascular system and systemic reactions (Wallace et al., 2007; Guo et al., 2012; Kang et al., 2017; Zhang et al., 2018; Van Dingenen et al., 2019). Among NSAIDs aspirin is the most widely used drug in the aged population for different reasons. Since 2013, Antibe Therapeutics Inc. is developing H_2_S-releasing NSAIDs, in particular ATB-340, which is a novel H_2_S-releasing derivate of aspirin (H_2_S-aspirin, **Figure 1**) but its effect on age-related changes of gastric mucosal defense system against high fructose injury is unknown. Therefore, in this study, we compare effects of H_2_S donors—NaHS, on the CBS, CSE, TST expressions in adult, and aged rats, as well as during treatment by ATB-340 in aged rats exposed to high fructose diet (HFD) during 4 weeks in both aspirin- and stress-related models of gastric injury.

## Materials and Methods

### Animals, Diets, and Treatments

All experiments were carried out on male adult (age = 12‑14 weeks) and aged Wistar rats (age = 42‑46 weeks) (n = 62) following the norms of animal experiments that guaranteed by ARRIVE guidelines and EU Directive 2010/63/EU for animal experiments and the local animal care committee at the Danylo Halytsky Lviv National Medical University Ethics Committee (protocol № 6 from 29.03.2017). All efforts were made to minimize animal suffering and to reduce the number of animals used.

### Experimental Protocol

**Figure 2:**
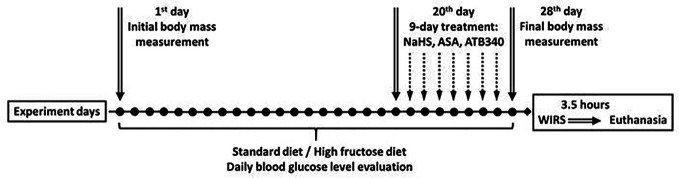
Design of the experiment.

Animals were maintained under a constant 12-h light and dark cycle and an ambient temperature of 21-23° C with 50 ± 10% relative humidity. All animals were kept in raised mesh-bottom cages to prevent coprophagy and subdivided into groups (n = 5‑6). **Figure 2** represents a schematic overview of the experimental protocol. Rats were randomly assigned to nine experimental groups. Rats in the control groups had free access to water and were kept on a standard diet (SD). Animals in the experimental groups received 28 days of fructose-supplemented water (HFD), unrestricted access to a 40% solution of fructose *ad libitum* (Bezpalko et al., 2015). Food deprivation for 12 h before the end of experiments has been performed for all rats. The initial and ﬁnal body weights in all animals were recorded by an RN 10C13U, 100 g-10 kg, ± 5 g (Vaga, Kyiv, Ukraine).

The rats were deeply anesthetized with an intramuscular injection of ketamine (60 mg/kg; Biovet, Bila Tserkva, Ukraine), sacrificed and after that, the stomachs were resected. Later stomachs were cut open along the lesser curvature and rinsed with saline and were taken for histological examination.

The animals were subdivided into control groups of adult rats and aged rats with SD and vehicle (1.0 ml of saline), and experimental groups receiving 28 days hypercaloric HFD, without and with acute stress. Acute stress was induced on the 29^th^ day of study by the model of Takagi and Okabe, 1968, that involves short-term exposure to water-immersion restraint stress (WIRS) (Takagi and Okabe, 1968). The rats were placed in restraint cages and immersed vertically to the level of the xiphoid process in a water bath of 23° C for 3.5 h. The initial and ﬁnal body weights were recorded. Blood glucose concentrations were measured daily by glucometer using a blood sample from the tail vein, as described previously (Zayachkivska et al., 2014).

Fasted rats from experimental groups were pre-treated 9-days (from 20^th^ to 28^th^ day of study) intragastrically by: 1) saline, as vehicle (adult); 2) vehicle (aged); 3) NaHS, 5.6 mg/kg/day (adult); 4) NaHS, 5.6 mg/kg/day (aged); 5) vehicle with stress induction (adult); 6) vehicle with stress induction (aged); 7) NaHS, 5.6 mg/kg/day and induction of stress; 8) ASA, 10.0 mg/kg/day and NaHS, 5.6 mg/kg/day and stress induction (aged); 9) ATB-340 17.5 mg/kg/day and induction of stress (aged). The administration of NaHS and ATB-340 was performed in doses tested previously (Zayachkivska et al., 2014; Wallace et al., 2015).

### Chemicals and Drugs

Sodium hydrosulfide (NaHS), D, L-homocysteine 3.3 mM, L-cysteine, Na_2_S•9H_2_O were obtained from Sigma-Aldrich (St. Louis, MO, USA). Pyridoxal phosphate 0.67 mM, N,N-Dimethyl-p-phenylenediamine sulfate, 99% was obtained from Acros Organic (New Jersey, USA).

Compound ATB-340 was obtained from Antibe Therapeutics (Toronto, Canada). ASA was purchased from Borshchahivskiy CPP (Kyiv, Ukraine).

All other chemicals used in the experiments were graded by the analytical method that was validated according to the guidelines of the International Conference on Harmonization (ICH).

### Morphological Evaluation of Gastric Damage

The stomachs were opened along the lesser curvature. The severity of GM lesions was evaluated by macroscopic inspection and GM damage index using histological double-blind studies of two investigators. Gastric tissues were then fixed in 10% formalin, dehydrated, and embedded in paraffin wax. Paraffin sections of 5 mm were cut and stained with hematoxylin and eosin. Histological changes were checked under a microscope (Leica DM 750/4 and digital camera Leica DFC 420, Germany). The GM lesion, including epithelial cell damage, vasocongestion, hemorrhage, and inflammation, was measured by microscopy investigation, and the total injury in one stomach was assessed by GM damage index scoring that conducted using the criteria in **Table 1** expending four fields of view at 40 × magnification. The mean score was determined for each treatment group and results were expressed as GM damage index.

**Table 1:** Criteria used for semi-quantitative analysis of gastric mucosa damage.

Criteria	Score
The state of the epithelial layer on the degree of alteration	1—no changes; 2—desquamation of epithelium; 3—destruction of gastric foveoli; 4—hypertrophy and vacuolization of mucosa cells; 5—erosion
The state of subepithelial layer	1—no changes; 2—diffuse swelling of the submucosal basis; 3—pronounced uneven swelling of the submucosal base and insigniﬁcant inﬁltration; 4—severe swelling and disorganization of the submucosal basis; 5—perivascular hemorrhages
The epithelial layer leukocyte inﬁltration	1—no leukocyte inﬁltration; 2—moderate leukocyte inﬁltration; 3—average leukocyte inﬁltration; 4—severe leukocyte inﬁltration; 5—multiple leukocyte inﬁltrates

The measurement of GM damage was determined by protocol-blinded researchers. The number of animals showing these histopathological lesions in each group was compared with that of other groups.

### Biochemical Analysis

Blood glucose concentrations were measured daily by glucometer (Achtung TD-4207, Munich, Germany) using a blood sample from the tail vein.

At the end of the study (on 29^th^ day), after euthanasia animals were sacrificed, the samples from rat gastric mucosa tissue were evaluated for the catalytic activities of CBS, CSE, and TST in H_2_S biosynthesis and oxidative stress markers. The stomach was washed with cold 1.15% potassium chloride solution, after which the mucous membrane was separated and homogenized in a medium of 1.15% potassium chloride in a ratio of 1:4. GM homogenates were centrifuged at 600 g and 40° C for 30 min to obtain a post-nuclear fraction.

### CBS, CSE, and TST Activities

We evaluated CBS, CSE, and TST activities in gastric tissue (nmol/min*1 mg of protein), using a modified version of the Stipanuk and Beck method (Stipanuk and Beck, 1982). Substrate and cofactor concentrations, pH, and incubation time, which could provide optimal conditions for enzyme activity determination, were selected in advance.

The activity of CBS was determined in the incubation medium containing in final concentrations pyridoxal phosphate 0.67 mM, L-cysteine 3.3 mM, L-homocysteine 3,3 мМ, Tris-HCl buffer 0.08 M (pH 8.5).

The activity of CSE activity was determined in incubation medium containing in final concentrations pyridoxal phosphate 0.67 mM, L-cysteine 3.3 mM, Tris-HCl buffer 0.08 M (pH 8.5).

The activity of the TST was determined in incubation medium containing in the final concentrations: sodium thiosulfate 0.2 mM, 1,4-Dithiothreitol 2.3 mM, Tris-HCl buffer 0.09 M (pH 8.5).

0,1 ml post-nuclear homogenates of the stomach (1:4 w/v in 1,15% Potassium chloride solution) were added to 0.5 ml incubation medium. After 60 min incubation at 37° С in sterile hermetically sealed plastic Eppendorf tubes (to prevent H_2_S losses), the reaction was quenched with cooling the tubes on ice and adding of a 0.5 ml 1% zinc acetate solution to bind the produced H_2_S.

The control samples were treated similarly, except the studied material was added to the medium after incubation and cooling. The amount of H_2_S was estimated with the methylene blue production by a standard method (Van Dingenen et al., 2019). There were added 0.5 ml 20 mM N,N-dimethyl-p-phenylenediamine in 7.2 M HCl, 0.4 ml 30 mM FeCl3 in 1.2 М HCl to the samples and incubated for 20 min at 18‑22° С, then added 1 ml 20% TCA and centrifuged for 10 min at 3000 rpm.

The optical density of the supernatant was measured at 670 nm in a cuvette with an optical path of 1.0 cm using the Apel PD-303 spectrophotometer (Japan). Sulfide anion content in the sample was calculated using a calibrated graph (Zaichko et al., 2009).

### Calculation of the Oxidative Index

For oxidative injury assessment, the oxidative index, as the value of imbalance between pro- and anti-oxidative activity, was calculated using the principle developed by Vassale et al.; Shimomura et al. (Vassalle et al., 2012; Shimomura et al., 2017), as a ratio between the content of lipid peroxidation products and anti-oxidative activity. For biomarker of lipid peroxidation products, the malonic dialdehyde level was detected as demonstrated previously (Pavlovskiy et al., 2018) and for the anti-oxidative activity—the TST colorimetric activity, as reported earlier.

### Statistical Analysis

All results were evaluated using Statistical Analysis System and visualization program «Statistica 7.0» (StatSoft, Informer Technologies, Inc.) and expressed as mean ± standard deviation (SD) for a series of experiments. A paired Mann–Whitney U-test was used for comparisons of paired treatments between two groups, and one-way ANOVA using Dunnett’s test was performed to compare different experimental groups with control. Statistical significance was set to p values ≤ 0.05.

## Results

### Effect of 28 Days High Fructose Diet-Induced Injury on Body Weight and Glucose Levels, and Histologic Changes of Gastric Mucosa

**Figure 3:**
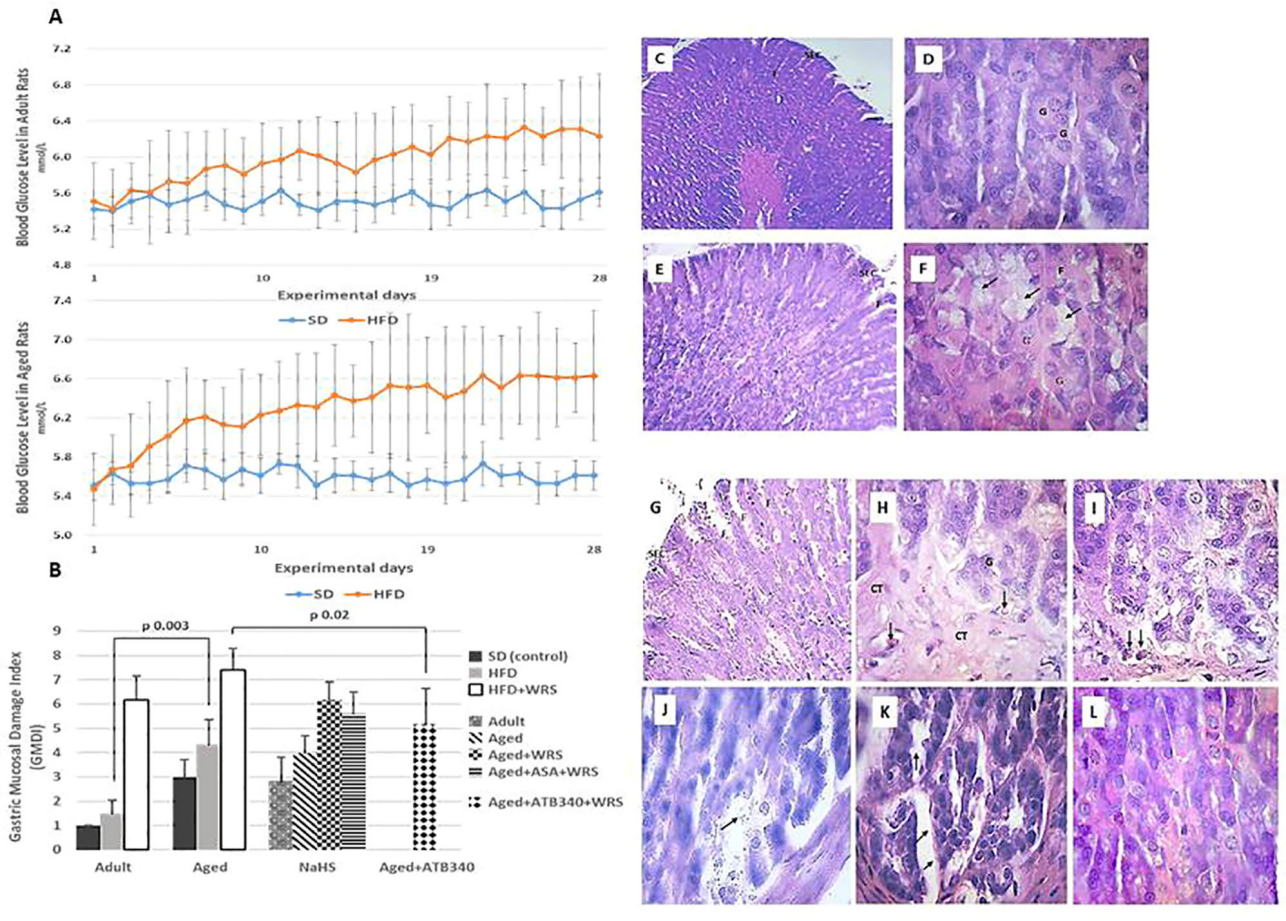
Blood glucose levels in adult (**A**, left graph) and aged (**A**, right graph) rats fed a standard diet (SD) or high fructose diet (HFD) during 28 days. Results are given as mean ± standard deviation (SD) (statistical analysis: one way-ANOVA, n = 5–6/group). Histological characteristic of study groups according to the changes in gastric mucosal damage index **(B)** and representative photomicrographs to illustrate the changes in the gastric mucosa in adult **(C, D)** and aged **(E–L**) rats fed by 28 days of high fructose diet, visualizing surface epithelial cells (SEC), foveoli **(F)**, and glandular cells **(G)**. Hematoxylin and eosin staining at low (C – x 150; E and G – x 200) and higher magnification (**D**, **F**, and **H–L** – x 600). Gastric mucosa of adult and aged rats shows glandular cells (**D**, **F**, respectively), and hypertrophy of mucous neck cells (arrows) **(F)**. In the gastric mucosa of vehicle-treated aged rats exposed to acute stress **(G, H)** and aspirin pretreatment **(I),** there is observed connective tissue (arrows) in basal third of the gastric mucosa, hyperemia in lamina propria (arrows). The gastric mucosa of aged rats exposed to acute stress and pretreated by NaHS **(J)**, a combination of NaHS and aspirin **(K)** shows uneven swelling of the submucosal base in gastric mucosa (arrows) while pretreatment of ATB-340 **(L)** shows diffuse swelling of the submucosal basis of gastric mucosa.

Rats consuming drinking water supplemented with fructose for 28 days exhibited a significant elevation of blood glucose levels (from 5.5 ± 0.4 mmol/L to 6.2 ± 0.7 mmol/L—adult rats; from 5.5 ± 0.3 mmol/L to 6.6 mmol/L—aged rats; p < 0.05) (**Figure 3A**), and about 10% gain in body weight (339 ± 31 g—adult rats; 499 ± 34 g—aged rats) over that of the control rats with SD (306 ± 21 g—adult rats; 458 ± 28 g—aged rats).

The adult and aged rats fed by SD and treated with vehicle exhibited normal gastric macroscopic appearance. The data in **Figure 3B** shows that the GM damage index of adult control rats on SD was 1.00; in aged rats on both SD and HFD, the GM damage index was 3 times higher, respectively, vs. adult rats (p < 0.05). Representative photomicrographs of gastric mucosa in adult and aged rats fed by 28-days of high fructose diet, visualizing surface epithelial cells, foveoli (pits), and glandular cells demonstrated **Figures 3C–L**. The differences of gastric mucosa of aged rats with the glandular cells and hypertrophy of mucous neck cells were observed (**Figures 3E, H**) in comparison to the adult rats (**Figures 3C, D**). Examination of the gastric mucosa of adult rats on HFD revealed an increased GM damage index in 1.5 times vs. the control group of adult rats (p > 0.05).

To assess the adaptive changes of GM defense in aged rats fed by HFD and treated by H_2_S donors exposition to WIRS was used. In this experiment, the histological changes of GM in aged rats treated with vehicle without and with exposition to stress were characterized by increased GM damage index in about 4 and 7, respectively, over the adult rats group on HFD treated with vehicle was 1,5 (p < 0.05), (**Figure 3B**). In the gastric mucosa of aged rats fed by HFD, vehicle-treated and exposed to acute stress (**Figures 3G, H**), we observed connective tissue in the basal third of the gastric mucosa, hyperemia in lamina propria (arrows) and hypertrophy and vacuolization of glandular cells (arrows). Similar changes of gastric mucosa were observed in the rats with aspirin pretreatment (**Figure 3I** **).**

In the gastric mucosa of aged rats exposed to acute stress and pretreated by NaHS (**Figure 3J**) and with combination of NaHS and aspirin (**Figure 3K**) we identified uneven swelling of the submucosal base in gastric mucosa (arrows) while pretreatment of ATB-340 (**Figure 3L**) shows diffuse swelling of the submucosal basis of gastric mucosa.

Treatment of ATB-340 attenuated gastric injury and resulted in 1.4 times lower GM damage index compared to rats treated with vehicle (p < 0.05). There was also observed decreased susceptibility of GM by lower GM damage index in the ATB-340 treatment group than in the group with co-treatment by the combination of NaHS and ASA group, as well as vehicle group regarding to the difference of GM damage index (p > 0.05).

### CBS, CSE, TST Activities, and Oxidative Index

**Figure 4:**
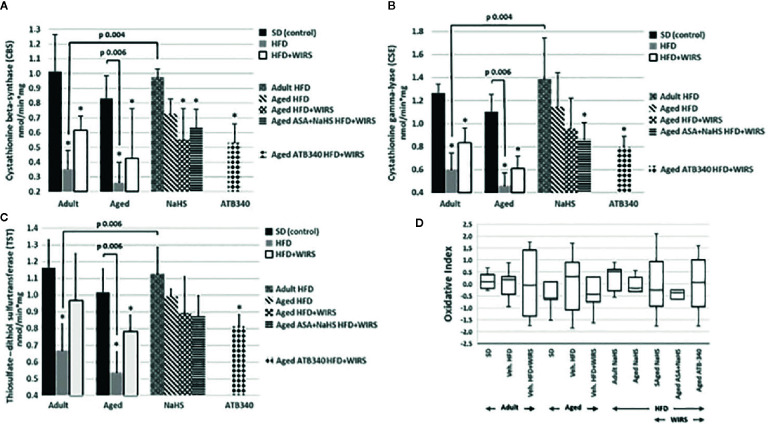
Activities of Cystathionine-β-synthase (CBS) **(A)**, Cystathionine-γ-lyase (CSE) **(B)** and Thiosulfate-dithiol sulfurtransferase (TST) **(C)** and changes of the oxidative index **(D)** in adult and aged rats (n = 5–6) fed with standard diet (SD) or high fructose diet (HFD) without and with H_2_S releasing molecule compounds therapy (NaHS, ATB-340) and induction of acute stress (WIRS). Columns with whiskers **(A, B, C)** show mean ± standard deviation (SD). Box plots **(D)** show the median, lower, and upper quartile ranges, and the minimum and maximum values of the oxidative index of all groups; *p < 0.05 versus control.

To evaluate the role of CBS, CSE, and TST activity in H_2_S signaling during HFD-induced GM malfunction in rats, in this study, we examined changes in their activities (**Figures 4A–C**), and oxidative index (**Figure 4D**). In intact adult control rats on SD, the activities of CBS, CSE, TST were reaching 1.01 ± 0.25 nmol/min*1 mg of protein, 1.26 ± 0.08 nmol/min*1 mg of protein and 1.16 ± 0.16 nmol/min*1 mg of protein, respectively. In groups on SD, there were decreased enzyme activities in aged rats compared to adult rats (**Figures 4A–C**). Besides, there was a significant difference in enzyme activities between adult and aged rats on HFD versus SD. Adult rats on HFD had much lower activity of CBS—35%, CSE—48%, and TST—56%, compared to SD group (p < 0.05). Aged rats on HDF have lower activities of CBS, CSE, and TST versus aged rats on SD; however, we observed increased activities of all H_2_S-related enzymes during induction WIRS (**Figures 4A–C**). We found, that in adult rats exposed to stress on HFD and treatment by NaHS resulted in increased activities of CBS—1.8 times, CSE—1.3 times, and TST by 69% over adult rats with vehicle (p < 0.05). Adult rats on HFD with NaHS administration exhibited a higher activity of CBS by 35% than aged rats (p < 0.05). However, the co-treatment of aged rats by NaHS and ASA was alike H_2_S–ASA, causing a similar activity of CBS (p < 0.05).

Given these results, we determined the oxidative index. We analyzed data of the age-related changes of the oxidative index in **Figure 4D**. It has shown that the oxidative index is increased in aged over adult rats fed by SD or HFD. However, the rats fed by HFD have higher levels of the oxidative index than animals fed by SD. The pretreatment by H_2_S donors (NaHS and ATB-340) in aged rats reduced the oxidative index levels and correlate with TST activity, suggesting that treatment demonstrates an anti-oxidant effect.

## Discussion

According to the recent data, among the most important risk factors for the increased susceptibility of GM are hyperglycemia and aging which could be triggers for inflammaging and metaflammation—immune-metabolic age-related conditions that contribute to manifestation of cytotoxic injury (Qi et al., 2012; De Cabo and Diaz-Ruiz, 2020). Several animal and human studies suggest that postprandial hyperglycemia related to excessive fructose consumption causes cellular and subcellular oxidative damage, and accelerates the age-related decline in cytoprotection by developing oxidative damage related to a modification in H_2_S-NO cross-talk and redox homeostasis (Guan et al., 2012; Cao and Bian, 2018). The process of age-associated increased susceptibility to gastric cytotoxic factors involves several vascular associated molecular signaling pathways, which has a bi-directional detrimental role on the gastric mucosal defense (Yonezawa et al., 2007; Velázquez-Moyado et al., 2018). It has been shown that the key role in gastroprotection at the advanced age belongs to the endothelial dysfunction, reduced mucosal defense, amplified susceptibility to injury by a variety of injurious agents and its dramatic consequences on older patients with hyperglycemia could be expected. ASA is one of the most common drugs among NSAIDs that are commonly reported as gastrotoxic agents, bringing hemorrhagic erosions and bleeding which is seriously threatening human health, especially in older persons. An increasing body of evidence confirms that using new H_2_S-releasing compounds, which increase endogenous H_2_S content by catalytic activities in H_2_S signaling, exert gastrointestinal safety by anti-oxidative, anti-inflammatory, and vasoactive effects (Dombkowski and Russell, 2004; Zhang et al., 2018; Qin et al., 2019; Pereira-Leite et al., 2020). H_2_S is important for redox regulation of multiple processes, including regulation of mitochondrial function through its effects as an electrons donor for the electron transport chain and inhibition of mitochondrial permeability transition pore (MPTP) opening, mitochondrial oxidation, and reaction producing thiosulfate (Donnarumma et al., 2011; Zhang et al., 2017; Mys et al., 2018), as well as the proper functioning of the immune system (Wallace et al., 2012; Qin et al., 2019). Therefore, our study was designed to test the hypothesis that linking H_2_S-related physiological mechanisms in gastroprotection and H_2_S-based therapeutic strategy for reduction of malfunction in gastric mucosal defense in advanced age and chronic nutritional carbohydrate overload. In this study we have used the experimental model of HFD, which mimics the modern “obesogenic” environment on rats and compare age-related effects on the gastric mucosa of 9-day exogenous administration of H_2_S donors: the inorganic sulfide salt—NaHS, the commonly used compound as a positive control in animal preclinical studies, which rapidly increases content of plasma H_2_S level, co-treatment of NaHS with conventional ASA and novel NSAID_S_ hybrid ASA-releasing H_2_S. To test adaptive changes of gastric mucosa in feature of age-related changes in gastroprotection, we used aged rats fed with HFD and exposed to WIRS. This animal model mimics early signs of the preclinical appearance of gastric ulcerogenesis observed in older patients who use the nutritional overload by unhealthy carbohydrates. Also, we determined the effects of H_2_S donors, such as NaHS and ATB-340 on CSE and CBS expression, which play a crucial role in H_2_S biogenesis, as well as TST expression which appears exclusively mitochondrial and has co-operative activity with mitochondrial ribosomal proteins L18 (MRPL18) and acts as a mitochondrial important factor for anti-oxidative stress functions and redox regulation, producing thiosulfate and recovering glutathione for defense cellular thiol proteins under oxidative stress (Szabo et al., 2014; Kimura, 2019). We defined that 4 weeks fructose nutritional overload decrease gastric mucosal defense in aged rats in contrast to adult animals and similar up-toward trend for age-associated results was in rats fed with SD and accompanied by increased GM damage index and modulated activities of CBS, CSE, and TST and changes of the oxidative index, reflecting the shift to oxidative damage. In addition, we identified decreased gastric CBS and CSE activities in HFD aged rats over adult rats. During exposure to stress, the activity of H_2_S-related enzymes CBS, CSE, and TST increased in contract to animals without stress, as the result of response to acute injury, nonetheless level of the oxidative index also increased in aged rats fed by HFD. These signs of oxidative damage not only negatively affect membranes, but also could initiate the mitochondrial process of apoptosis, which results in cellular damage due to MPTP opening and re-store NO-synthase coupling (Nakajima, 2015; Mys et al., 2018; Kimura, 2019). These results indicate that the H_2_S pathway is likely to be an important determinant of gastric mucosa susceptibility to HFD injury in advanced age. Our data of CBS, CSE, and TST activities correlate with histological results that exogenous administration of H_2_S donors reverses GM damage that results on H_2_S-NO cross-talk (Zhang et al., 2017; Cao and Bian, 2018), sirtuin-1 (SIRT1) activity that has the ability for increasing mitochondrial function, protects against oxidative stress and can promote angiogenesis, as well as H_2_S-dependent protein persulfidation (Arumugam and Kennedy, 2018). We analyzed the data during treatment by H_2_S donors that characterized by decreased oxidative index and could conclude that in our model of HFD injury the increased CSE, CBS, and TST activities during exposure to stress could be explained by additional involvement of bioavailability of H_2_S despite oxidative index changes which exist probably due to the effects of aging and HFD. This is a significant finding because these types of effects could be mechanisms contributing to age-related changes in gastroprotection. This effect was mediated by activation of endogenous H_2_S-associated compounds, which have free-radical scavenging activity. Our data could be explained by previous results of several scientific groups that show the role of sulfhydryl compounds in gastric cytoprotection (Szabo et al., 1981; Wallace et al., 2007). When we compare age-related effect of NaHS on gastroprotection we detected twice lower level of the oxidative index in adult animals over the level of aged rats. Moreover, the NaHS-mediated treatment decreased CBS and CSE activities twice, as well as TST activity by 30% in aged rats over the results of adult rats. A similar trend was obtained during co-administration of NaHS with ASA and resulted in attenuating gastric damage induced by HFD and aging-associated changes in redox balance. Interestingly, the administration of ATB-340 was accompanied by a more intensive decrease of CSE, CBS, and TST activities than during co-treatment with NaHS and convention aspirin. This observation remains in agreement with recent data from several studies during chronic inflammatory conditions when conventional and H_2_S-releasing compounds reduced oxidative stress in different tissues decreasing the process of lipid peroxidation, myeloperoxidase activity in a different way, however, only H_2_S-NSAID_S_ showed increased glutathione levels in the tissue and reduced plasma extravasation and gastrointestinal damage and could be promised compounds to decrease susceptibility to oxidative stress (Kang et al., 2017; Ajayi et al., 2018). Thus, data of TST expression, in aged rats with pretreatment by H_2_S donors during HFD with WIRS, and GM damage index could be explained by their ability for intracellular antioxidant activity possessing which ameliorates mitochondrial dysfunction that, in turn, improves respiratory electron transport. It helps to explain our obtained results of the gastroprotective effect of ATB-340, as protection against oxidative stress injury of the gastric mucosa resulted in the decreased oxidative index during exposure of aged rats to WIRS.

In conclusion, the H_2_S pathway is essential reason of gastric mucosa susceptibility to HFD injury in advanced age and administration of exogenous H_2_S donors has demonstrated the efficiency against the reduction of age-associated gastric mucosal defense. Decreased activation of H_2_S signaling in the HFD model might be related to the increased mitochondrial malfunctions and oxidative stress that promote excessive TST expression. Gastroprotection during ATB-340 (H_2_S-ASA) treatment may be relevant to the increased activity in H_2_S signaling by attenuation of oxidative stress.

### Translational Perspective

Pharmacological intervention to increase endogenous H_2_S levels extends anti-oxidative activity suggesting that H_2_S donors could be promising ingredients for the new strategy to treat age-related and HFD-induced malfunctions. Further studies are needed to study the pharmacologically dose-dependent activities of H_2_S donors NaHS, and H_2_S-ASA and exact molecular mechanisms to explore their anti-oxidative activity. H_2_S-ASA has the potential for the target-oriented treatment of gastric malfunction related to HFD and age-associated changes.

## Data Availability Statement

The raw data supporting the conclusions of this article will be made available by the authors, without undue reservation.

## Ethics Statement

The animal study was reviewed and approved by animal care committee at the Danylo Halytsky Lviv National Medical University Ethics Committee (protocol № 6 from 29.03.2017).

## Author Contributions

OZ designed the study. YP performed experiments and data analysis. Gastric histological evaluation was done by YP and AY. YP, AY, and OZ interpreted the findings and prepared and completed the manuscript. All authors contributed to the article and approved the submitted version.

## Funding

This work has been supported in part by Danylo Halytsky Lviv National Medical University under the project “Role of systemic and local mechanisms in cytoprotection under the extreme influence” (the state registration ID 0116U004510).

## Conflict of Interest

The authors declare that the research was conducted in the absence of any commercial or financial relationships that could be construed as a potential conflict of interest.

## Abbreviations

ASA, Acetylsalicylic acid; ATB-340, H_2_S-aspirin; CBS, Cystathionine beta-synthase; COX, Cyclooxygenase; CSE, Cystathionine gamma-lyase; FeCl_3_, Iron (III) chloride; GM, Gastric mucosa; GPx, Glutathione peroxidase; GSH, Glutathione; GSR, Glutathione-disulfide reductase; H_2_S, Hydrogen sulfide; H_2_S-NSAIDs, Hybrid H_2_S-releasing drugs, analogs of nonsteroidal anti-inflammatory drugs; HCl, Hydrogen chloride; HFD, High fructose diet; K^+^ ATP-channels, Adenosine triphosphate-sensitive potassium channels; MPTP, Mitochondrial permeability transition pore; MRPL18, Mitochondrial ribosomal proteins L18; NaHS, Sodium hydrosulfide; NFκB, Nuclear factor kappa-light-chain-enhancer of activated B cells; NO, Nitric oxide; NSAIDs, Nonsteroidal anti-inflammatory drugs; ROS, Reactive oxygen species; SD, Standard diet; SIRT1, Nicotinamide adenine dinucleotide-dependent deacetylase sirtuin-1; TLR4, Toll-like receptor 4; TST, Thiosulfate-dithiol sulfurtransferase; WIRS, Water immersion restraint stress.
